# Exploring perceptions of cannabis use and employment implications among healthcare workers: a single-institution experience

**DOI:** 10.1186/s42238-026-00399-8

**Published:** 2026-02-06

**Authors:** Axel B. Lichtenberg, Ramy El Mankabady, Monica Mansour, Annette Wang, Catherine Wagner, Curt Bay, Frank Bauer

**Affiliations:** 1 William Carey University College of Osteopathic Medicine, 710 William Carey Pkwy, Hattiesburg, MS 39401 USA; 2 HonorHealth Osborn Medical Center, 7351 E Osborn Road Suite 200B, Scottsdale, AZ 85251 USA; 3 A.T. Still University, 5850 E Still Cir, Mesa, AZ 85206 USA

**Keywords:** Healthcare workers, Cannabis use, Attitudes, Survey, Research

## Abstract

**Background:**

Cannabis use is becoming increasingly prevalent in the United States, and its implications extend into many facets of society, including healthcare. Despite legal and societal shifts toward the acceptance of cannabis, healthcare professionals face ethical and legal complexities related to cannabis use. Understanding perceptions of cannabis use within the healthcare community is essential for developing informed drug policies in healthcare settings. The objective of this study was to better understand the changing perceptions of recreational and medical cannabis use on employment within the healthcare community.

**Methods:**

In September 2023, a team of residents and faculty conducted an anonymous descriptive survey study targeting residents, attending physicians, and administrative staff at HonorHealth in the Phoenix metropolitan area (Arizona, USA). The survey included questions on participants’ roles in the healthcare community, their knowledge of cannabis and cannabis testing policies, and their opinions on the permissibility of cannabis use in various workplace scenarios. The survey results were summarized via descriptive statistics.

**Results:**

The survey received 80 responses from 258 potential respondents, yielding a 31% response rate. The survey indicated familiarity among respondents with cannabis products, support for removing cannabis from pre-employment screening, and disapproval of employment termination following positive tests, particularly for medical cannabis use.

**Conclusions:**

The findings suggest that healthcare institutions need more information about cannabis use in these environments and highlight areas for policy review and further research to inform evidence-based approaches to drug policies that balance ethical considerations and the well-being of healthcare professionals.

**Supplementary Information:**

The online version contains supplementary material available at 10.1186/s42238-026-00399-8.

## Background

Cannabis use has experienced a dramatic surge across the United States in recent years, with over 64 million Americans reporting using cannabis in 2024 (Substance Abuse and Mental Health Services Administration 2024). This significant uptake follows the rapidly evolving legal landscape, where recreational use is now permitted in numerous states alongside widespread medical cannabis programs. This societal shift raises essential questions about cannabis use within professional contexts, particularly in healthcare environments where there is a complex intersection of patient safety concerns, professional standards, and legal obligations (Ryan et al. 2021). Understanding perceptions of cannabis use within the healthcare professional community is important for developing informed drug policies in healthcare settings as well as driving accurate research related to cannabis use in this population.

While some studies have reported that cannabis use among nurses and physicians is comparable to that of the general population (Naillon et al. 2023; Rainbow et al. 2024), cannabis use among healthcare professionals remains understudied. Healthcare professionals operate in a uniquely challenging position of legal ambiguity with cannabis use as institutional policies are often zero-tolerance, even in states with legalized recreational access (Ryan 2021, Perlman et al. 2021). This environment creates substantial barriers to accurate research, as healthcare workers may justifiably fear professional censure, licensure consequences, or career limitations if they disclose cannabis use (Abramowitz 2020), even for research purposes.

Unless a state expressly protects the use of medical cannabis, employees may have no protection against termination or other disciplinary action (Boehmer 2021). However, this stance is subject to change as societal attitudes toward cannabis continue to evolve and more states enact protection for cannabis users. As of 2025, 24 states have legalized adult-use cannabis, with nearly half of these laws enacted after 2020 (National Conference of State Legislatures 2024). Combined with the United States government’s recent move to reschedule marijuana (Friedman et al. 2024), these developments highlight the need to reassess how both medical and recreational cannabis use impact employment in the healthcare sector. The extant literature was published either prior to noteworthy changes in cannabis legislation (Raskin 1993; Donohoe 2005; Lemon et al. 1992) or only focused on attitudes towards the use of cannabis or its legalization (Jacobs et al. 2022; Makki et al. 2022; Weisman and Rodriguez 2021), leaving a gap in understanding how these changes in beliefs affect employment among healthcare stakeholders.

Our study aimed to address this critical knowledge gap by investigating the understanding and attitudes toward both recreational and medical cannabis use as they specifically relate to employment considerations among stakeholders in the healthcare professional community. By examining perceptions across different roles and specialties, we aimed to identify emerging consensus points that can inform more evidence-based employment policies in this rapidly changing environment.

## Methods

### Study overview

This study was conducted within the HonorHealth (HH) Graduate Medical Education (GME) program in the Phoenix metropolitan area (Arizona, USA). The target population included resident physicians, fellows, and attending faculty affiliated with HH training programs. A total of 258 individuals were identified from GME program distribution lists and invited to participate. Before commencing data collection, on August 10, 2023, we received Institutional Review Board (IRB) exemption status from the HH IRB (Study #: IRB-23-008).

### Study design and recruitment

The research team designed a 32-question electronic survey to explore perceptions of cannabis use and its implications for employment. Survey content was based on a review of relevant literature (Raskin 1993; Donohoe 2005; Lemon et al. 1992; Jacobs et al. 2022; Makki et al. 2022; Weisman and Rodriguez 2021; Bell et al. 2015; Pham et al. 2015) and discussions with stakeholders in the HH community to ensure relevance and content validity. An expert panel reviewed the draft instrument to assess face and content validity prior to distribution. The final survey included multiple-choice items, Likert scale ratings, and open-ended comment sections to support both quantitative and qualitative analysis. The survey was administered online via a secure platform, accessible through a link which was emailed to the target population. Participation was voluntary and anonymous, with electronic informed consent obtained at the beginning of the survey. Respondents could skip any question; missing responses were handled using pairwise deletion, and no imputation was applied.

Eligible participants included all residents, fellows, and attending physicians affiliated with HonorHealth GME programs. Individuals outside this group or those who declined informed consent were excluded. Survey invitations were emailed to all 258 individuals on September 4, 2023, using program contact lists. Two reminder emails were sent, and resident champions were asked to promote survey participation at education meetings. As an incentive, participants could opt into a drawing for one of three $100 gift cards to local restaurants. The survey remained open through October 4, 2023.

### Study outcomes

The primary outcomes of the survey included respondents’ familiarity with cannabis products, (e.g., knowledge of legality and pharmacology, such as whether cannabis use is federally legal or whether THC causes euphoria) awareness of cannabis testing policies, and opinions on employment related drug screening (e.g., Likert scale ratings of agreement with statements such as “Residents should be screened for marijuana use before employment” and whether termination should follow a positive test, particularly in cases of medical cannabis use). The survey also explored respondents’ attitudes toward cannabis use in professional healthcare settings using case-based scenarios, where respondents evaluated whether healthcare professionals using medical or recreational cannabis should be allowed to continue employment. Details of all survey questions and their responses can be found in the provided supplemental data (Supplemental Data S1 – Cannabis Survey Questions, Supplemental Data S2 – Cannabis Survey Data).

### Analysis

**Fig. 1:**
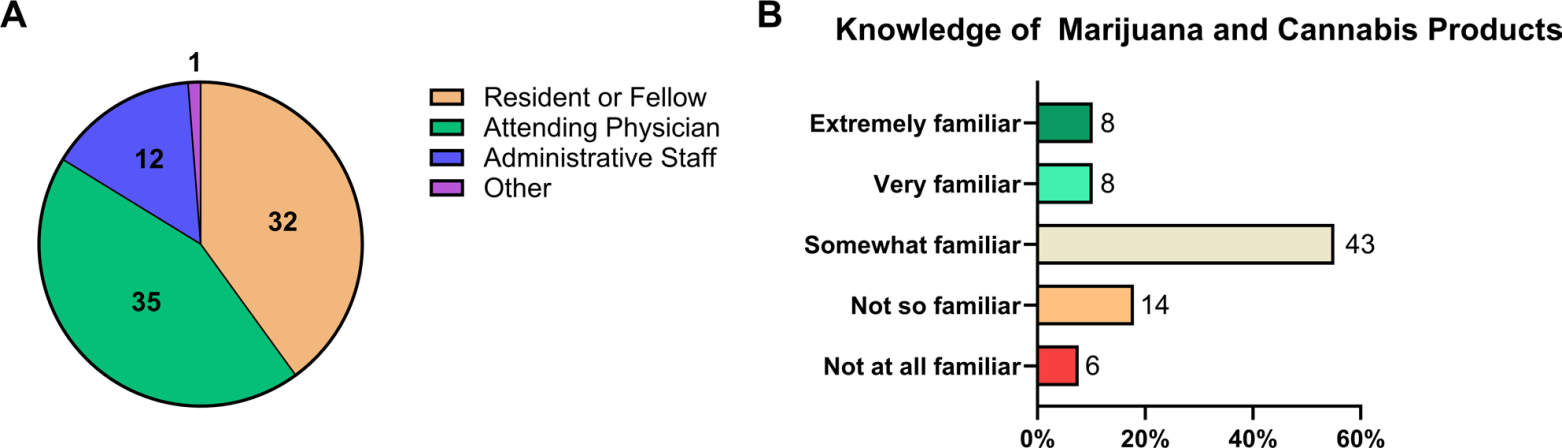
Respondent Population and Knowledge of Marijuana and Cannabis Products.** A** Summary chart of respondents by profession, total *n* = 80. **B** Reported respondent knowledge of marijuana and cannabis products, ordered by “extremely familiar”, “very familiar”, “somewhat familiar”, “not so familiar”, and “not at all familiar”, total *n *= 80

Quantitative data were analyzed using descriptive statistics, including percentages and frequencies for multiple-choice questions. For qualitative responses, we used a team-based review. Two authors independently read the open-ended comments, identified recurring themes, and then discussed them together until they reached agreement on how to summarize the responses in a way that best represented the participants’ perspectives. No formal coding software or predefined coding schema was used, but this collaborative process helped ensure that the identified themes accurately reflected the participants’ views. Assuming that approximately 258 employees met eligibility requirements, responses from 81 participants would yield a confidence level for responses of 95% ±10%. Responses to case scenarios were also compared across different participant roles. SPSS Statistics v. 28.0 (IBM Corp., Armonk, New York, USA) was used for statistical analysis and GraphPad Prism v 10.4.0 (Dotmatics, Boston, Massachusetts, USA) was used to present the data in Fig. 1.

## Results

### Respondent summary

Responses were received from 80 out of 258 potential respondents, demonstrating a 31% response rate. Among these participants, 40% identified as residents or fellows, 43.8% as attending physicians, 15% as administrative staff, and 1.3% fell into the ‘other’ category (Fig. 1A). There was no statistical difference in responses when compared by role. Most respondents reported undergoing pre-employment drug screening during their hiring process, and the majority of respondents (75.6%) indicated being at least “somewhat familiar” with cannabis products (Fig. 1B). A portion of participants (46.25%) reported an unawareness of HH cannabis testing policies (Supplemental Data S2 – Cannabis Survey Data, question 3). Overall, responses were split regarding removal of cannabis screening for residents/fellows/faculty, but leaned toward support for removal of pre-employment cannabis screening more generally and toward disapproval of employment termination policies following positive tests, particularly in cases involving medical cannabis use (41.6% strongly disagreed, 36.4% disagreed; Table 1).

**Table 1:** – Opinions on cannabis screening and employment termination policies [n (%)]. Responses to Likert-scale questions assessing healthcare professional's attitudes toward cannabis screening and employment termination policies, including pre-employment screening and termination following positive cannabis tests, with skipped responses noted

Policy Aspect	Strongly Disagree	Disagree	Neutral	Agree	Strongly Agree	Total (*n*)	Skipped (*n*)
Mandatory cannabis screening for employment of residents/fellows/faculty	14 (18%)	19 (24.3%)	14 (18%)	19 (24.3%)	12 (15.4%)	78	2
Employment termination after a positive cannabis test in residency or fellowship training	21 (26.9%)	23 (29.5%)	14 (18%)	13 (16.7%)	7 (8.9%)	78	2
Termination with a medical cannabis card	32 (41.6%)	28 (36.4%)	13 (16.8%)	4 (5.2%)	0 (0%)	77	3
Termination with FDA-approved treatment	25 (32.5%)	32 (41.5%)	10 (13%)	8 (10.4%)	2 (2.6%)	77	3
Exclude cannabis from pre-employment screenings	6 (7.8%)	13 (16.9%)	17 (22%)	22 (28.6%)	19 (24.7%)	77	3

### Awareness and knowledge of cannabis use

Respondents reported familiarity with cannabis products (10.26% reporting being extremely familiar, 10.26% very familiar, 55.13% somewhat familiar, 17.95% not so familiar, 7.69% not at all familiar with marijuana and cannabis products, Fig. 1B). They were highly aware of the legal status of both medical and recreational cannabis use in Arizona and accurately identified the effects of THC and the non-euphoric properties of CBD (Supplemental Data S2 – Cannabis Survey Data, questions 7–8, 10–11).

### Screening policies and employment

Most respondents supported exclusion of cannabis from screening for pre-employment (24.7% strongly agreed, 28.6% agreed with excluding cannabis from screening; Table 1). Responses regarding whether residents/fellows/faculty specifically should undergo pre-employment cannabis screening were split, with substantial proportions both supporting and opposing screening (Table 1). The majority disagreed with policies that would lead to employment termination after a positive drug test for cannabis, especially in instances where employees possessed a medical cannabis card (Table 1). Responses to specific case-based scenarios revealed varying levels of support from the majority (> 50%) for continued employment based on the circumstances of cannabis use (Table 2), including 72% support for continued employment of an attending physician following a positive drug screen (Supplemental Data S2 – Cannabis Survey Data, question 31).

**Table 2:** – Summary of responses for Case-Based scenarios. Summary of responses to hypothetical case-based scenarios involving healthcare workers using recreational or medical cannabis. Percentages represent the proportion of respondents who were in favor of continued employment (i.e., not terminating the employee) given the context of each scenario, with qualitative themes summarized in the Results column

Case #	Scenario	Percent	Results
1	28 y/o female Medical Assistant failed gold standard tx for refractory spasticity symptoms and self-prescribed a THC and CBD regimen.	64.4%	There was no statistical difference by healthcare role, with more than half of the respondents supporting continued employment. Additional comments included the need to measure potential impairment objectively regarding direct patient care.
2	54 y/o male Nurse uses Epidiolex for refractory seizures approved for children by the FDA.	75.3%	There was no statistical difference by healthcare role, with three-quarters of the respondents supporting continued employment. Additional comments included the need for objective measurement of potential impairment.
3	30 y/o female Family Medicine resident on dronabinol (synthetic form of THC) for chemotherapy-induced nausea and vomiting.	82.4%	There was no statistical difference by healthcare role, with most respondents supporting continued employment. Some comments suggested adjusting clinical duties or considering medical leave for medication-related impairments.
4	General Surgery resident on dronabinol under similar conditions.	62.7%	There was no statistical difference by healthcare role, with slightly over half of the respondents supporting employment. The lower support for continued employment may be due to the skill involved with surgery and comments called for objective impairment measurement and possible adjustments to duties.
5	34 y/o attending physician screens positive for recreational marijuana during post-offer employment drug testing.	60%	There was no statistical difference by healthcare role, with slightly over half of the respondents supporting employment. There were comparisons made to alcohol, and the focus should be on preventing impairment rather than punitive measures.

## Discussion

This descriptive study revealed a complex variety of opinions and attitudes toward cannabis use in professional healthcare settings that both align with (Martins et al. 2022; Bell et al. 2015; Philbrick et al. 2020; Rønne et al. 2021; Weisman and Rodriguez 2021) and diverge from (Phillips et al. 2015; Yang et al. 2024; Zwerling et al. 1990) findings in the prevailing literature.

Most individuals (*n* = 41, 53.3%) surveyed supported excluding cannabis from pre-employment screening, and had critical views on employment termination following positive cannabis tests, which contrasts with earlier more strict calls for drug testing (Phillips et al. 2015; Yang et al. 2024; Zwerling et al. 1990). This sentiment aligns with a growing societal shift toward the decriminalization and legalization of cannabis (Martins et al. 2022) and reflects an evolving understanding of cannabis use within professional contexts. The opposition to employment termination policies following a positive drug test, particularly in cases involving medical cannabis cards, suggests a call for more nuanced, compassionate approaches to drug policies in healthcare settings. When presented with specific case-based scenarios involving healthcare workers and cannabis use, respondents consistently favored nuanced approaches, “as long as use of marijuana doesn’t impair her ability to perform her clinical responsibilities”, focusing on observable impairment and job performance, rather than blanket termination policies (Supplemental Data S2 – Cannabis Survey Data, question 32). This preference for contextualized assessment over strict prohibition suggests that these healthcare professionals view cannabis through a harm-reduction lens rather than a prohibitionist framework. Some respondents compared cannabis-related policies to those governing alcohol, “if the detectable level can be established that demonstrates a person non-impaired (like the 0.08% for drivers) then I think it is fine”, and “we do not screen for…. alcohol. We should not do so for marijuana” (Supplemental Data S2 – Cannabis Survey Data, questions 31–32), noting the broader need for consistency in addressing legalized substance use.

However, the absence of a widely accepted standardized test to detect impairment from cannabis complicates this approach, as THC metabolites remain in the bloodstream long after intoxication (Huestis 2015), making it a poor indicator of impairment (Arkell et al. 2021). Although researchers have identified ratios of active compounds to metabolites as potential indicators of recent marijuana consumption (Kosnett et al. 2023), further research is needed to develop tests that can rapidly and accurately assess workplace intoxication. In addition to chemical testing, non-chemical assessment tools such as cognitive and psychomotor performance measures are being developed to detect cannabis-related impairment. For example, the DRUID (Driving Under the Influence of Drugs) mobile application has been studied as a potential method to assess real-time impairment through reaction time and divided attention tasks (L’Heureux 2019). These emerging approaches may complement biochemical assays in providing a more practical and accurate measure of workplace intoxication.

This survey revealed knowledge and awareness gaps among respondents regarding cannabis products and the consequences of positive cannabis tests, which aligns with findings from previous studies (Bell et al. 2015; Philbrick et al. 2020; Rønne et al. 2021). The findings from this study highlight the ongoing need in the healthcare community for education about cannabis-related employment policies, the legal landscape surrounding cannabis, and the use of medical cannabis while protecting patient safety. By increasing awareness of cannabis and related policies, healthcare institutions can potentially reduce issues related to employment termination, prompt changes to outdated policies, and improve patient care. As research in this field continues, the development of a reliable test to assess cannabis impairment may soon become available, potentially addressing some of these challenges.

Limitations of this study include the small sample size and geographic focus limited to one healthcare center, which may restrict the generalizability of the presented findings. Although our response rate appeared low at 31%, we note that some non-responses may be due to inactive email addresses, spam filtering, or infrequent email use. The study captures opinions only at a single point in time, and attitudes toward cannabis use are likely to continue to change over time. Finally, the survey may not have covered all aspects of cannabis use and employment policies relevant to the healthcare community, potentially limiting the scope of our findings.

Future research should include broader demographic and geographic representations and develop reliable impairment assessment tools for cannabis use in healthcare settings. Longitudinal studies could provide insight into evolving perceptions of cannabis use and its impact on professional performance and patient care outcomes.

## Conclusion

These survey results from the HH healthcare community provide insight into how evolving public consensus surrounding cannabis use can be integrated into professional practice. The respondents supported moving away from punitive measures toward more nuanced, performance-based assessments. The findings reflect the attitudes of respondents within a single healthcare system and should be interpreted as a preliminary view into evolving perceptions, rather than a broad representation of norms across the healthcare industry. Additionally, institutions may wish to explore investing in the piloting and validation of impairment assessment tools, both biochemical and performance-based, as these approaches could provide a more consistent basis for policy development. Establishing research-based guidelines and developing reliable impairment assessment tools for cannabis use in healthcare settings would support the creation of policies that ensure both patient safety and fair treatment of healthcare professionals.

## Supplementary Information

Supplementary Material 1: Cannabis Survey Questions.

Supplementary Material 2: Cannabis Survey Data.

## Data Availability

All relevant data are provided within the manuscript or supplemental data files.

## Abbreviations

HH: HonorHealth
IRB: Institutional Review Board
