# Supplementary material for: Exploring perceptions of cannabis use and employment implications among healthcare workers: a single-institution experience
Source: J Cannabis Res. 2026 Feb 6;8:36. doi: 10.1186/s42238-026-00399-8 (PMC12973884; doi:10.1186/s42238-026-00399-8)
Supplement: Supplementary file 1 — Supplementary Material 1: Cannabis Survey Questions. [file 42238_2026_399_MOESM1_ESM.docx]

**Perceptions of cannabis use in the GME community**

Thank you for taking the time to complete this survey. The data gathered by this survey will be used to write an editorial piece that summarizes current perceptions of employment screening for marijuana use and examines whether it is a helpful or harmful hiring practice in the current GME climate. Your participation in this voluntary and anonymous pre-assessment is highly appreciated.

**Role in Academic Affairs**

GME administrative staff

Faculty (any status)

Resident or Fellow

**Current Hiring Practices at HH**

For each question choose the one option that best matches your opinion. You are not required to answer every question.

1. During the hiring process, were you asked to complete a pre-employment drug screen? (yes/no)
2. To your **knowledge**, is **marijuana included in the drug screen** for pre-employment intake at HonorHealth? (yes/no)
3. To your knowledge, a **positive urine** drug screen (UDS for marijuana at HonorHealth can **lead to a loss of employment**? (yes/no)
4. To your knowledge, a **positive urine** drug screen for marijuana at HonorHealth can **result in rescinding a pre-employment** **offer**? (yes/no)

**Knowledge of marijuana and cannabis products**

1. How would you rate your knowledge of marijuana and cannabis products. (1-5)
2. To your knowledge, marijuana use is medically legal in the state of Arizona. (true/false)
3. To your knowledge, marijuana use is recreationally legal in the state of Arizona. (true/false)
4. To your knowledge, marijuana use is federally legal. (true/false)
5. To your knowledge, CBD, a cannabinoid of marijuana, creates a euphoric effect. (true/false)
6. To your knowledge, THC, a cannabinoid of marijuana, creates a euphoric effect. (true/false)
7. To your knowledge, there are no FDA approved uses of marijuana. (true/false)

**Is marijuana screening important?**

5-point Likert response sets - Rate how much you agree or disagree with the following statements:

1. Residents/fellows/faculty should be screened for marijuana use before employment.(1-5)
2. Medical students should be screened for marijuana use before rotations. (1-5)
3. Staff members not directly involved in patient care should be screened for marijuana use before employment. (1-5)
4. Use of marijuana determined by positive urine drug screen should jeopardize employment during residency or fellowship training. (1-5)
5. Use of marijuana determined by positive urine screen should jeopardize employment for staff members not directly involved in patient care. (1-5)

**Are there circumstances where a positive screen is appropriate?**

5-point Likert response sets - Rate how much you agree or disagree with the following statements:

1. A positive marijuana urine drug screen should lead to termination of employment, even if the employee has a medical marijuana card. (1-5)
2. A positive marijuana urine drug screen should lead to termination of employment, even under special conditions such as FDA approved treatments, if the employee does not have a medical marijuana card. (1-5)

**Should marijuana screening be discontinued?**

5-point Likert response sets - Rate how much you agree or disagree with the following statements:

1. Marijuana should (remain) be removed from the list of substances screened for pre-employment. (1-5)
2. Residency programs should conduct their own pre-employment screening for recreational marijuana use even if the institution does not. (1-5)

**Marijuana and Physician Impairment**

1. In your opinion, how much does physician use of marijuana affect patient care? (1-5)
2. How likely are you to suspect an impaired physician if an adverse event were to occur after this policy reversal (if this policy was reversed)? (1-5)
3. Should impairment due to marijuana be defined by a specific level of THC detected (e.g., five nanograms or more per milliliter in whole blood)? (1-5)
4. Pre-employment drug testing detects and prevents drug abuse. (1-5)
5. Random drug testing detects and prevents drug abuse. (1-5)

**Case Scenarios**

28 yo female with multiple sclerosis with refractory spasticity symptoms failed the gold standard of treatment. She is a medical assistant at a local doctors office. She can perform her job duties with the spasticity symptoms, but they make it harder for her to be efficient in her work. After speaking with her neurologist, she decides to take an oral formulation that contains a combination of THC and CBD. Of note, there is a higher dosage of THC than CBD in this formulation. Do you think this woman should be allowed to take this formulation of medical cannabis and still be allowed to continue in her position work? (yes, no, unsure, blank space to comment)

54 yo male with history of refractory seizures even with the gold standard treatment modality. He works as a nurse on the telemetry floor at a community hospital. When he is not having seizures he is able to perform his job without issue. After speaking with his neurologist, he elects to start a medication called edpidiolex which is a CBD containing liquid taken orally. He does note that this is an FDA approved treatment for seizure disorders in children and is hoping this will also help reduce his seizures, although it has not been studied in his age group. Do you think he should be allowed to take this formulation of medical cannabis and be allowed to continue in his position? (yes, no, unsure, blank space to comment)

30 yo female family medicine resident who is going through chemotherapy for a newly diagnosed lymphoma. Unfortunately, she develops chemotherapy induced nausea and vomiting that does not respond to the gold standard anti-nausea medications or is a contraindication due to QT prolongation. After speaking with her oncologist, she decides to start a medication called dronabinol, which is an oral capsule that contains THC. Through her own research, she did discover that this medication is FDA approved for treatment of nausea, vomiting, and cachexia. Do you think this woman should be allowed to take this formulation of medical cannabis and still be allowed to continue in her position? (yes, no, unsure, blank space to comment)

If she were a general surgery resident, would you feel the same way?

(yes, no, unsure, blank space to comment)

60 yo male who is an air force war veteran and currently flies commercial airlines presents with refractory PTSD and anxiety that does not respond to the gold standard treatment modality. After speaking with his psychiatrist, he elects to start a CBD containing pill that has <0.3% THC to help with his symptoms. The psychiatrist explains to this gentleman that CBD does not have the psychoactive effects of THC. Do you think he should be allowed to take this formulation of medical cannabis and still be allowed to continue in his position? (yes, no, unsure, blank space to comment)

43 yo attending physician who works in the state of Arizona where recreational marijuana use is legal. They choose to unwind after a long week by smoking recreational marijuana or taking an edible during their day off. The next week this physician is asked to perform a randomized urine drug screen (UDS). Their UDS returns positive for marijuana. Should this physician be allowed to continue work? (yes, no, unsure, blank space to comment)

34 yo physician has applied to a fellowship and an offer was extended to invite her to this fellowship. The physician is asked to take a pre-employment urine drug screen per policy. She tests positive for marijuana and nothing else. Should this fellow’s employment offer be retracted? Why or Why not? (yes, no, unsure, blank space to comment)
